# Microbiomes for All

**DOI:** 10.3389/fmicb.2020.593472

**Published:** 2020-11-12

**Authors:** Theodore R. Muth, Avrom J. Caplan

**Affiliations:** ^1^Department of Biology, Brooklyn College of The City University of New York, Brooklyn, NY, United States; ^2^Molecular, Cellular, and Developmental Biology Department at The Graduate Center of The City University of New York, New York, NY, United States; ^3^Department of Biology, Dyson College of Arts and Sciences, Pace University, New York, NY, United States

**Keywords:** undergraduate research, microbiology education, big data, data analysis, microbiomes, course-based undergraduate research

## Abstract

Microbiome research projects are often interdisciplinary, involving fields such as microbiology, genetics, ecology, evolution, bioinformatics, and statistics. These research projects can be an excellent fit for undergraduate courses ranging from introductory biology labs to upper-level capstone courses. Microbiome research projects can attract the interest of students majoring in health and medical sciences, environmental sciences, and agriculture, and there are meaningful ties to real-world issues relating to human health, climate change, and environmental sustainability and resilience in pristine, fragile ecosystems to bustling urban centers. In this review, we will discuss the potential of microbiome research integrated into classes using a number of different modalities. Our experience scaling-up and implementing microbiome projects at a range of institutions across the US has provided us with insight and strategies for what works well and how to diminish common hurdles that are encountered when implementing undergraduate microbiome research projects. We will discuss how course-based microbiome research can be leveraged to help faculty make advances in their own research and professional development and the resources that are available to support faculty interested in integrating microbiome research into their courses.

## Introduction

The study of microbiomes has skyrocketed over the last decade and has advanced our understanding of human health and disease, complex ecological systems, microbial diversity, and evolution (Falkowski et al., 2008; Locey and Lennon, 2016; Thompson et al., 2017; Almeida et al., 2019). The use of the term, microbiome, has jumped from fewer than 5 publications/year prior to 2008, to more than 6,000 publications/year in 2019, and microbiome studies have been the focus of numerous news and internet stories (Bik, 2016; Schmulson and Bashashati, 2018; Abid, 2019). Growth in microbiome research has been driven in part by new DNA and RNA sequencing and analysis technologies, and by a paradigm shift in the field of microbial ecology, sparked by culture-independent techniques (we will use culture-independent to include both metagenomics, *sensu stricto*, and gene-targeted amplicon sequencing) (Handelsman, 2004; Riesenfeld et al., 2004; Escobar-Zepeda et al., 2015; Goodwin et al., 2016; Slatko et al., 2018). These changes created an opportunity to bring the excitement and potential of microbiome studies to students through training in the scientific process and their engagement in research (Jurkowski et al., 2007; National Research Council, 2007). This review discusses microbiome research in teaching microbiology to students at two intersections with the real-world, (1) the ability to advance understanding in areas of human health and disease, biodiversity, evolution, biotechnology, climate science, and other fields, and (2) the increasingly in-demand skills of quantitative reasoning, statistics, and data skills (mining, analysis, interpretation and visualization), and the spectrum of STEM classroom and laboratory contexts in which students receive their training. As a target of exploration in STEM education, microbiomes capture our imagination with their complexity, ubiquity, and potential to contribute solutions to global health and environmental crises (Blaser et al., 2016; Finbow, 2019). For educators, the versatility of microbiome studies as a scaffold for teaching microbiology, ecology, evolution, genetics, bioinformatics, and data analysis, is unmatched.

Microbiome research projects are ideal for teaching microbiology in a real-world context. Importantly, large microbiome data sets can be generated and analyzed in a massively parallel fashion by students working individually or in small groups (Hingamp et al., 2008; Boyle, 2010; Buonaccorsi et al., 2011; Bolyen et al., 2019). Students are excited by work on unanswered questions and take ownership of research projects that make use of samples they have collected in their communities and local environments (Lopatto, 2010; Hanauer and Dolan, 2014; Weber et al., 2015; Cooper et al., 2019a). Culture-independent approaches do not require growing microorganisms in the lab. As a result, this work poses few safety risks to students and allows microbiome research in almost any classroom setting and expands the reach of these research projects to citizen science initiatives (Freeman et al., 2016; Handelsman et al., 2018; American Society for Microbiology, 2019; Genné-Bacon and Bascom-Slack, 2019; Basalla et al., 2020). Because of the flexibility and the range of questions that can be addressed, microbiome studies open avenues to interdisciplinary research that extend across courses, departments, and institutions. The instrumentation required for culture-independent studies of microbial diversity does not need to be extensive, making these projects accessible to many high school, community college, and public university faculty (Estes, 2015; Robertson-Albertyn, 2016). Investigating and analyzing microbiome data is ideal for training in quantitative reasoning, data analysis, and data presentation. While this list of strengths associated with implementing student microbiome projects is significant, there are also significant hurdles, and these vary depending on the background and expertise of faculty and the availability of resources. For those who have experience working with microbiomes, classroom and laboratory logistics and pedagogical considerations remain a primary challenge. For faculty that are veterans of undergraduate research experiences (UREs) and course-based research experiences (CUREs), but who are new to research into microbiomes, the fast-paced advances in sequencing and data analyses tools can be a challenge to keep up with and add an element of uncertainly to implementing microbiome projects.

In the literature searches for this review we found more than twenty published examples of undergraduate microbiome research projects (Table 1) that may serve as helpful aids for those looking for guidance on designing and structuring a course that includes microbiome research. The review gives examples of how the challenges of experimental design, data collection, and data analysis with students have been addressed by others. There are communities of faculty with experience in undergraduate microbiome research projects, such as the Research Experiences in Microbiomes Network (REMNet, an NSF RCN-UBE), and communities such as these can be an additional source for ideas and support for developing student microbiome research projects. Using a range of UREs, including CUREs, guided inquiry, capstone research projects, intensive summer research experiences, and other modalities, we and others have developed resources for faculty and students to explore the diversity and complexity of their local environments using microbiome research projects. The tools for studying microbial communities, and for DNA or RNA sequencing and data analysis, are increasingly accessible and affordable, and they can extend the reach of UREs into cutting-edge applications. Moreover, hands-on experiences addressing real-world questions are an important part of training of the next generation of STEM professionals for a society where complex scientific and technological skills will be critical (Kloser et al., 2011; Vision and Change in Undergraduate Biology Education, 2011; Auchincloss et al., 2014).

**TABLE 1:** Microbiome research projects for undergraduate students.

References	Topic	Audience	Discipline
Boomer et al., 2002	Green non-sulfur bacteria of Yellowstone NP	Upper-level UG	Molecular biology
Hingamp et al., 2008	Metagenome annotation	Upper-level UG	Cell biology and Biochemistry
Rios-Velazquez et al., 2011	Soil microbiomes	Upper-level UG	Microbiology
Donato et al., 2012	Identification of a reductase gene	Upper-level UG	Biochemistry
Edwards et al., 2013	Marine microbiomes	Upper-level UG	Ecology, multidisciplinary
Muth and McEntee, 2014	Urban microbiomes	Introductory and upper-level UG	Microbiology, Intro-biology
Rundell et al., 2014	Winogradsky columns	NS	Microbiology
Sanders et al., 2016	Plant microbiomes	Upper-level UG	Biology
Docherty et al., 2015	Soil microbiomes	NS	Multidisciplinary
Gibbens et al., 2015	Mississippi River water samples	Introductory UG	Biology
Muterspaw et al., 2015	Mixed environmental samples	Upper-level UG	Ecology, Computer Science
Wang, 2017	Oral microbiomes	Upper-level UG	Biology, Biotechnology, Microbiology
Hartman et al., 2016	Personal microbiomes	Introductory UG	Bioinformatics
Coil et al., 2017	Gut microbiome board game	Introductory UG	Microbiology
Costas et al., 2017	Influence of fertilizer on nitrogen-fixing microorganisms	Introductory HS	Biology (HS)
Hotaling et al., 2018	Prokaryotic diversity	Introductory UG	Biology
Lentz et al., 2017	Human umbilicus microbiomes	Introductory and upper-level UG	Biology and Biotechnology
Stevens et al., 2017	Environmental microbial communities	Upper-level UG	Microbiology
Alessi et al., 2018	Soil microbiomes using isolation chips (iChips)	Upper-level UG/MS	Molecular microbial ecology
Scott Weber et al., 2018	Personal microbiomes	Upper-level UG	Immunology, Molecular Biology, Genomics
Tobin and Shade, 2018	Effect of temperature on soil bacteria	Upper-level UG	Microbiology
Skendzic and Keler, 2019	Fruit fly gut microbiome	Introductory UG	Biology
Cottone and Yoon, 2020	Leaf microbiomes	Introductory UG	Biology
Goller and Ott, 2020	Swab samples	Upper-level UG and Grad	Biotechnology
Parks et al., 2020	Winogradsky columns, urban microbiomes, kombucha	Introductory and upper-level UG	Microbiology, Microbial ecology
Riley et al., 2020	Gold-precipitating bacteria (*Delftia* spp.)	Introductory and upper-level UG	Interdisciplinary

*Searching the available literature, this table lists articles that describe student microbiome research projects. The list is not likely to be exhaustive as other articles that fit the criteria may have been missed if the search terms were not present in the title, abstract, or key word fields. There are many other articles that describe excellent bioinformatics and genomics research projects but they are not included here if they did not explicitly mention a microbiome element to the research project. Abbreviations: UG = undergraduate, MS = masters, Grad = graduate, HS = high school, NS = not specified.*

## Advancing Scientific Knowledge Through Microbiome Research

The opportunity for significant impacts from microbiome research has been made possible by the confluence of emerging forces – the culture-independent study of microbial communities, the power and accessibility of next-generation DNA sequencing and analysis tools, and the push to provide research experiences to more students (Handelsman, 2004; Escobar-Zepeda et al., 2015). The pioneering work on 16S rRNA genes by Carl Woese and the initial culture-independent studies of bacteria in the mid-80s and 90s by Norman Pace, Jo Handelsman and others, have invigorated and revolutionized the field of microbiology (Gutell et al., 1994; Riesenfeld et al., 2004; Frank and Pace, 2008). Prior to these breakthroughs, little of the microbial world could be coaxed to grow under laboratory conditions, and often less than 1% of the diversity from samples could be studied. This was hinted at by the discrepancy between the diversity of bacteria seen by microscopy and the relative lack of diversity in what could be cultured in the lab from the same sample (the “great plate count anomaly,” Staley and Konopka, 1985). Culture-independent approaches, which use the extractable DNA as a proxy for the microorganisms present in a sample, now allow investigators to routinely study > 95% of the diversity in a sample – thus opening up new paths of discovery and insight into how the world’s most numerous and influential cells (and viruses) are shaping the environment (Blaser et al., 2016). The advances have a far-reaching impact on critical research and development areas, including drug discovery, agriculture, environmental sustainability, and ecosystem resilience in the face of anthropogenic forces such as urbanization and climate change (Nielsen et al., 2012; King, 2014; Barea, 2015; Harvey et al., 2015; Jez et al., 2016; Brown and Hazen, 2017; Krüger et al., 2018; van de Guchte et al., 2018; Podolsky et al., 2019; Xie et al., 2019).

Following on the heels of culture-independent approaches to studying microbiomes, the first wave of next-generation DNA sequencing instruments became available to biologists in the mid-2000s (Goodwin et al., 2016; Slatko et al., 2018). This catapulted the depth of DNA sequence analysis from the few hundred reads that could be generated through clone libraries and Sanger sequencing, to hundreds of millions of reads generated by next-generation sequencing instruments. Together, culture-independent approaches and next-generation sequencing allow the study of complex, dynamic, microbial systems in greater detail than was previously possible. This work has led to the discovery of new phyla and has greatly increased our estimates of the diversity of bacteria, fungi and viruses in environments across the globe (Venter et al., 2004; Hug et al., 2016).

## Teaching Quantitative Reasoning and Data Skills Through Microbiome Research

**FIGURE 1:**
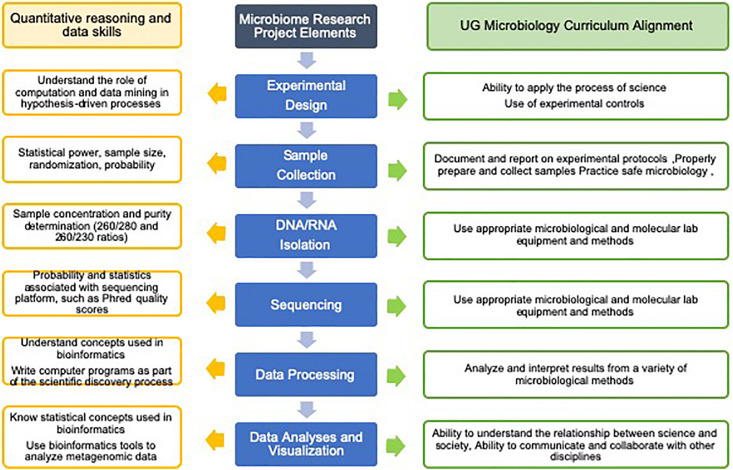
Microbiome research projects can be designed to meet specific curriculum goals and to include quantitative reasoning and data skills. This figure illustrates how the basic elements of a standard microbiome research project can be aligned with the curriculum and specific data and analysis skills. Additional microbiology curriculum details and quantitative reasoning and data skills can be found in the references, American Society for Microbiology (2012) and Wilson Sayres et al. (2018).

Research experiences with microbiomes provide training for students in essential quantitative reasoning and data analysis skills that can be applied in the field of microbiology and many other STEM fields, as well as in non-STEM professions that are reliant on large and complex data sets (Chapman et al., 2006; Tan et al., 2009; Nelson and Campbell, 2011; Fenlon, 2017; Mulder et al., 2018; Attwood et al., 2019). There have been a number of recent reports that anticipate the needs of the research community and define the skills and experience students should have in order to enter research fields. A 2016 study surveyed more than 700 NSF principal investigators from the Biological Sciences Directorate (NSF-BIO) and asked where they saw unmet needs for analyzing “big data” for biological research (Barone et al., 2017). The top two categories of unmet data analysis needs were “training on integration of multiple data types” and “training on data management and metadata,” and topping the list of major data types used by these PIs were DNA/RNA/protein sequence data. The Network for Integrating Bioinformatics into Life Sciences Education (NIBLSE) Core Competencies Working Group developed a set of 15 bioinformatics skills for undergraduate life sciences students and analyzed survey responses from 1260 biologists (Wilson Sayres et al., 2018). Their findings show the skills receiving the highest score (“extremely important” on a Likert-scale) include, “understand the role of computation and data mining in hypothesis-driven processes within the life sciences,” “know statistical concepts used in bioinformatics,” “know how to access genomic data,” and “be able to use bioinformatics tools to analyze genomic data.” The study included a comparison of the relative importance of the bioinformatics skill and evidence that the skill was addressed in course syllabi, and found that statistics and metagenomics skills were among those with the greatest disparity between importance and actual representation in course syllabi (Wilson Sayres et al., 2018). In addition, NIBLSE surveyed biology faculty asking what they perceived as barriers to the integration of bioinformatics into undergraduate courses and categorized frequently cited barriers into six categories (Williams et al., 2019). Three categories: Faculty Issues, Student Issues, and Curriculum Issues, were the most cited by biology faculty, and within these categories, specific barriers included a lack of faculty expertise and a lack of faculty time, students’ lack of background skills and students’ lack of basic computing knowledge, as well as insufficient availability of bioinformatics lesson plans and the rapid rate at which bioinformatics material changed (Williams et al., 2019). These barriers are not easy to overcome, but might be reduced through the integration of microbiome research into courses (Cline and Prokop, 2018). Microbiome research allows students to work with large data sets, to learn statistical analyses, and to provide insight through data interpretation (references cited in Table 1). Several elements of the ASM curriculum guidelines, *Concepts and Statements* (American Society for Microbiology, 2012), can also be addressed through microbiome research projects. All of the ASM guidelines’ *Scientific Thinking Skills*, which include, the “ability to apply the process of science,” the “ability to use quantitative reasoning,” and, the “ability to understand the relationship between science and society” can be addressed in microbiome research projects (Figure 1).

## Microbiome Projects in UREs

Considering the training that benefits young scientists, and the skills that will be required in the biological research workforce, how can undergraduate microbiome research projects help to provide training and meet curriculum standards? Early work with students amplified genes of interest from total DNA isolated from environmental samples and sequenced clone libraries to identify previously undescribed bacteria (Boomer et al., 2002). This provided insight into the diversity of bacterial communities and employed bioinformatics tools such as BLAST and tree-building programs, but it did not generate the amount of data that easily led to quantitative and statistical analyses. The introduction of next-generation sequencing, in combination with culture-independent studies, however, released a flood of microbiome sequence data and created an opportunity for students to contribute to the analysis. Using Global Ocean Sampling data sets, Hingamp et al. (2008) developed a parallel workflow to analyze reads from these data and students participated in “Annotathons” using bioinformatics tools to detect open reading frames and conserved domains, run BLAST searches and multiple sequence alignments, and construct phylogenetic trees (Rusch et al., 2007; Hingamp et al., 2008). Nearly 90% of the successfully classified sequences from the student Annotathons were bacteria, with an additional small percentage from Archaea and viruses. The student Annotathon process involves supervision and iteration to work toward a reliable sequence annotation, and is similar to models for student analysis of genomes and other biological data sets that had been developed earlier (Goodner et al., 2001; Hatfull et al., 2006; Slawson et al., 2006; Pico et al., 2008; Boyle, 2010; Ditty et al., 2010). Beyond sequence analysis, an ambitious ecological metagenomics course was developed for upper-level undergraduates and graduate students to contribute to on-going research of California sea lions and included surface marine water and kelp forest microbiome samples (Edwards et al., 2013). This interdisciplinary course took students through the process from DNA library preparation, next-generation sequencing, and data analysis, with student survey results demonstrating post-course acquisition of valuable wet-bench and data analysis skills. After initial “proof of concept” reports of investigating microbiomes with undergraduate students, a number of other publications followed that involved student studies of soil microbiomes, urban and built environment microbiomes, river microbiomes, plant and insect microbiomes, human microbiomes, and others (for details, see Table 1). While most CUREs involve learning practical laboratory skills as well as computational and bioinformatic skills, this is not always necessary. For example, Lentz et al. (2017) studied learning outcomes of a dry-lab approach using an open-access bioinformatic tool for analysis of human umbilical cord microbiomes. The positive learning outcomes included evaluating a hypothesis which is a skill usually associated with hands-on experimental design. The ability to engage students in data analysis research projects without a field or wet-bench component has been underscored by the current COVID-19 pandemic. In our experience, and from anecdotal reports of our colleagues, there are a number of undergraduate laboratory courses that have shifted focus to analyzing existing data sets with students online, and that have adapted approaches to meeting course learning objects that rely on remote, socially distanced, research experiences as a result of the constraints imposed by the pandemic. In parallel, we have observed an uptick in faculty demand for workshops and webinars that include emphasis on using data processing and analysis pipelines such as QIIME2 and mothur, as well as a desire to learn how to use more complex microbiome data analysis tools. These recent shifts underscore the flexibility of microbiome research and the potential to use microbiome projects as a scaffold for a range of learning objectives. While the social distancing measures necessitated by the pandemic will eventually be removed and allow faculty and students to resume in-person teaching, the impending budget crisis that many institutions will face is likely to require cost-cutting measures for years into the future. Shifting to microbiome data analysis with students, while not a perfect substitute for hands-on research experiences, could allow some courses to bridge a period of financial uncertainty by reducing the reagent and materials expenses that would be required for wet-bench labs.

## Accessibility – Reaching More Students With Microbiome Projects

Cost and accessibility are factors in the equation when deciding whether or not to incorporate a microbiome URE into the curriculum. Microorganisms grow quickly, respond rapidly to environmental change, are highly diverse, and often are inexpensive to grow and maintain, making them ideal for classroom use. Using culture-independent approaches allows many microbiome projects to be carried out safely in BSL-1 level labs, to be run in high schools, and meaningful research projects to be designed and executed by students. This enables students to engage in the process of science and foster project ownership while producing data sets that allow microbiological questions to be addressed using quantitative analysis skills.

Although the cost of DNA sequencing continues to drop, many microbiome projects can still come with a hefty price tag. It is, however, possible to run exciting microbiome projects with students and generate excellent data with costs that fit modest budgets. Considering the arc of a microbiome project, costs for soil, water, or swab sample collection and DNA extraction range from $5 to $10 per sample, reagents for PCR amplification and quantifying DNA are $5 to $8 per sample, shipping samples to a sequencing facility are $35 to $50 for overnight shipments, the sequencing costs themselves can be $50 to $100 per sample depending on the platform used and number of reads per sample, and finally, data analysis can be free, for basic taxa tables, to more than $100 per sample for detailed analyses. From our experience of using microbiome UREs in laboratory courses, we spend $150 to $300 on reagents and sequencing costs for 2–4 independent samples in a section of 18 students per semester. Savings associated with removing older and less effective elements from the laboratory curriculum allowed the inclusion of the microbiome UREs at almost no additional cost over what had been budgeted for the lab without microbiome UREs. These costs are based on a laboratory that was equipped for standard work with DNA, including pipettors, gel electrophoresis equipment, spectrophotometer, centrifuge, and thermal cycler, and no additional major equipment purchases were required.

An important strategy in keeping costs down is to have students work in small groups of three to six students, and to pool multiple DNA samples into a single sample for sequencing. In the analysis of complex and heterogenous environments, such as soil, it is recommended to collect a number of independent samples from a specified plot in order to accurately represent the microbial community (Knight et al., 2018). Having multiple student groups prepare independent DNA samples from the same site, and combining these samples into a composite sample for sequencing, both reduces costs and provides a better picture of the microbial community present at the sample site. For example, a lab of 18 students working in six groups of three can compare communities from two different sites, or compare an experimental treatment to a control, with each group preparing two DNA samples (for ∼$120) and sequencing only two composite samples (for ∼$120) is a savings of ∼$600 compared to the cost of sequencing each isolated DNA sample.

It is, however, an unfortunate reality that even a few hundred dollars can be too costly for some budgets, and if essential equipment is lacking, student microbiome projects can be pushed out of the reach of many classrooms. While the desired solution is that science education receives the funding that is required to train and prepare students in STEM disciplines, it is a fact that many public institutions, and institutions in underserved communities, simply are not funded adequately and they must turn to cost-cutting compromises. In surveys we have administered to faculty, the cost of microbiome projects is among the most significant barriers to their implementation as UREs. Working with our colleagues at REMNet (an NSF-funded RCN-UBE), it has been part of our mission to facilitate student microbiome research projects and to find creative ways to make projects accessible to as wide a range of classrooms as possible. In addition to the cost saving practices described above, REMNet has encouraged the introduction of research elements into the classroom through CUREs. Through “dual use” design, faculty can bring down the costs of CURE microbiome projects by aligning them with the goals of their own laboratory research, or through collaborations with research projects lead by investigators at their institution or nearby institutions. The coordination of CURE student research with faculty research projects can result in students’ projects contributing preliminary data for competitive grants aimed at supporting additional research. Professional development and training can also be significant costs for faculty that prevent the incorporation of microbiome projects into the curriculum. The REMNet community has been able to provide training and support for faculty through workshops, online videos, and a collection of protocols that have been developed for student microbiome projects. Finally, the overwhelming amount of data generated by many microbiome projects creates an opportunity for many downstream data analysis projects. For labs or courses without a wet bench component, or for those labs without access to essential equipment, it is possible to collaborate with other investigators who can provide sequence data to be analyzed by students. These shared data can serve as the basis of novel and demanding UREs that center on data analysis and data visualization. Through efforts such as those mentioned here, and other creative approaches, it is possible to make undergraduate microbiome research projects accessible.

Reports, such as Vision and Change, call for engaging students in the process of science and argue that greater engagement results in positive outcomes in student success, learning, problem solving, and an appreciation for research (Anderson et al., 2011; Vision and Change in Undergraduate Biology Education, 2011; Merkel, 2012). The default for undergraduate research is based on a faculty-mentored apprenticeship model, where students work on a project over a semester or longer. Students who engage in undergraduate research in this way show improved academic outcomes and greater levels of graduate school admissions (Cooper et al., 2019b; Nerio et al., 2019). A major limitation in the apprenticeship model, however, is that it is constrained by the number of faculty who can take on students in their lab. In many institutions, particularly community colleges and public colleges and universities, the potential demand for research experiences can exceed apprenticeship capacity by 10-fold or more (Parks et al., 2020). Accordingly, solutions have been sought that broaden student research training, such as intensive summer research programs and CUREs. While many CUREs have been developed recently, there is a track record of success, and some programs, such as the Superlab at Haverford College, have been training students using a CURE model for more than 50 years (Owen et al., 1991; Alkaher and Dolan, 2014).

Determining which mode of URE is the best fit for a microbiome research project requires coordination of the learning goals for students with the resources of time, funding, faculty expertise, and any parallel research objectives. A best practice in achieving this coordination is based on an incremental approach that begins with piloting a project on a small scale before moving on to incorporating a project into a large course, or courses with multiple sections and instructors. Small pilot projects provide an opportunity to identify potential problems in scaling-up and can produce initial data that are helpful in convincing colleagues and administrators that a larger microbiome project implementation is feasible. Traditional faculty-mentored research projects, small capstone courses, and summer research experiences often are formats that are well suited for the piloting phase. These formats can be stepping stones to larger and more ambitious microbiome CURE projects. As an example of this, the Authentic Research Experiences in Microbiology (AREM) program began with three undergraduate students working in a faculty lab as part of an independent research course studying urban microbiomes from city playgrounds, subway stations, and soil from local parks (Muth and McEntee, 2014). After adapting protocols for use with student groups and determining how the experiments would map onto the course schedule, the microbiome project was incorporated as a multi-week module in a single undergraduate microbiology lab section of 18 students (Biology majors in their 2nd or 3rd year of study). After two semesters in a single lab section, a second laboratory section was converted to include the AREM microbiome research module. At the same time a set of basic assessment tools were used that allowed a comparison of student learning and attitudes with microbiology lab sections being run at the same time using the traditional format without the AREM microbiome research module. The initial results showed that there was greater student engagement and excitement in the sections that included the microbiome research component, and gradually, the microbiome research module was incorporated into all ten laboratory sections over the next two semesters. Even with this step-wise approach, challenges remain, the most significant being the need to train additional instructors teaching the AREM module.

In addition to piloting microbiome research projects, it is helpful to start with well-defined projects with a narrow scope. Isolating and studying microbiomes from diverse natural, urban, and human environments has an inherent exploratory element that appeals to students, however, the study of microcosms in laboratory settings, such as Winogradsky columns, allows students to investigate how controlled experimental variables influence microbiomes (Rundell et al., 2014; Parks, 2015; Parks et al., 2020). Laboratory maintained microcosms are often better suited to comparative studies with proper controls and replication, and can be run in winter when collecting outdoor samples may not be an option. Akin to Winogradsky columns, in a modified AREM module, students use soil samples collected from the campus to test a hypothesis related to the competitive exclusion of potential pathogens. Each student group prepares three soil conditions, (a) one tube of 10 g of soil, (b) one tube of 10 g of soil spiked with a laboratory strains of *E. coli* and *S. epidermidis*, and (c) one tube of 10 g of autoclaved soil spiked with a laboratory strains of *E. coli* and *S. epidermidis*. Total DNA is extracted from 250 mg of soil from each tube on day one and again 3 weeks later. The multiple student groups provide the replication for this experiment. In a simple and inexpensive design such as this, students can develop and test a hypothesis, quantify the diversity and relative abundance of bacteria in a local soil sample, measure colonization resistance, see the limitations posed by the presence of relic DNA, as well as other questions. Controlled variables sometimes exist in the environment, as was the case for students studying the effect of soil temperature on bacterial communities isolated from above the Centralia, PA mine fire (Tobin and Shade, 2018), and fertilizer additions to agricultural soils (Costas et al., 2017). Selecting a question of interest, formulating a hypothesis, and developing an experimental design to test the hypothesis are important features of the process of science (Hoskins et al., 2011; Goldey et al., 2012; Smith et al., 2013). Including students in these steps can add to the value of a microbiome URE and can better target learning objectives than survey projects that study microbiomes from the environment without a guiding hypothesis.

There are many excellent examples of microbiology UREs that broaden participation and reach a demographic of students who might not otherwise engage in research (Eagan et al., 2013; Bangera and Brownell, 2014; Schinske et al., 2017). A number of large projects involve students in microbiology research on a national scale, including, Tiny Earth (Handelsman et al., 2018; Basalla et al., 2020), Small World Initiative (Hernandez et al., 2015; Davis et al., 2016), Prevalence of Antibiotic Resistance in the Environment (PARE) (Genné-Bacon and Bascom-Slack, 2019), Science Education Alliance-Phage Hunters Advancing Genomics and Evolutionary Science (SEA-PHAGES) project (Jordan et al., 2014), the Microbial Genome Annotation Network (MGAN), and AREM (Muth and McEntee, 2014). Having a diversity of options is useful because microbiology instruction occurs in many different contexts, with wide variations in faculty expertise, research interests, time availability, resources and infrastructure, student preparation, and curriculum requirements. While no single solution will suffice, microbiome research projects can be versatile and exciting, and are being used extensively in UREs, citizen science projects, and K-12 classes. Varying formats of microbiome research can be tailored to specific research and curricular goals. These include project-based, upper-level laboratory courses and intensive summer research programs where students self-select for participation and may have a number of pre-requisites to meet. Implementations such as these tend to be small sections with multiple experienced instructors who work closely with students to help them master complex sample preparation and sequencing protocols (Edwards et al., 2013; Rundell et al., 2014; Muterspaw et al., 2015). In a smaller class with expert support and sufficient access to computers, it is also feasible to carry out sequence filtering and processing using command line interface and pipelines such as QIIME2 (Bolyen et al., 2019). These are fantastic experiences for students and, with the appropriate design, they can generate publication quality data.

However, not every institution or department is able to run such a course, and even when possible, the smaller class size prevents many students from having an opportunity to participate before they graduate. Our experience with undergraduate microbiome research began at the City University of New York, the country’s largest urban university, and an environment that, because of its size and modest means, is only able to reach a small fraction of students with project-based, upper-level laboratory courses. We developed AREM as a flexible, modular microbiome research approach that faculty could integrate into existing courses and across multiple sections, without significant expense or logistical hurdles (Muth and McEntee, 2014). In most implementations, the modular AREM microbiome projects have a focus on microbiology content and less so on sequencing, data processing, and bioinformatics elements. It is important to align a microbiome research project with curricular goals, and in an introductory microbiology course it may be a better fit to emphasize microbial diversity, phylogeny, growth, competition, metabolism, and environmental influences, rather than devoting several sessions to the intricacies of data processing and advanced data analyses. In 16S amplicon sequencing-based projects, it is standard for most commercial and institutional sequencing facilities to provide a taxonomy table with relative abundances in a spreadsheet format, and this is often more than enough data for most courses, and precludes the need for student or faculty expertise using data processing pipelines. Using the AREM design, modular microbiome projects based on 16S sequence data sets have been used with high school students, at community colleges, at primarily undergraduate institutions, and at large universities. The core elements of experimental design, samples collection, DNA isolation, sequencing, and basic data analysis run through most implementations of modular microbiome projects and are easily adapted to fit specific course requirements, research goals, and learning objectives (Figure 1). Several experienced practitioners have worked together to establish the REMNet, and this network provides expert support and training resources to faculty working with students on microbiome research projects. The REMNet community expects to grow and develop, and to provide support for a wider range of amplicon sequencing, metagenomic, RNAseq, and other data types.

Looking beyond direct benefits to the scientific research community, a successful microbiome project should help students to develop technical expertise, and more generally, should help to develop their critical thinking skills and further their scientific understanding and ability to communicate their ideas. This is essential to the changing needs of the workforce that increasingly requires strengths in critical thinking, problem solving, and the ability to collaborate with colleagues. An important aspect of microbiome projects is engaging students by addressing questions without a predetermined outcome. A focus on unknown microbial communities excites and resonates with students because it involves the environments where they live, study, and work. The questions they ask, the data they collect, and the interpretations of those data are relevant to them and they are more likely to have a sense of ownership of the project. This engagement and project ownership can translate into a certain degree of pride and social responsibility, as students often talk about how they may have changed their own opinions, and the opinions of friends and family, in regards to the role of microorganisms in the environment and the connection to environmental and human health.

## Summary

Microbiome research projects are accessible to undergraduate students. From the development of research questions related to microbiomes, to DNA sequence analysis and data interpretation, students find themselves integrating information from the fields of genetics, ecology, statistics, epidemiology and health sciences. The involvement of interdisciplinary work underscores in the important collaborative nature of research and the need to exchange ideas and perspectives.

Microbiome studies may begin with unrelated research questions and different sampling sources, but the sequence output from most projects can be analyzed with a shared set of tools and allows for training in quantitative and data analysis skills. This underscores the versatility of microbiome studies in providing students with an opportunity to practice and learn from the exploration of large data sets using bioinformatics, data analysis, and data visualization tools. Microbiome research projects can be tied directly to questions on related to biodiversity and ecology, as well as topics that have social justice components such as climate change, food security, and human health. The relevance of these research areas to issues students encounter in the news and to issues discussed in public fora is attractive because it ties students’ academic studies directly to the real-world.

## Author Contributions

TM and AC contributed equally to the preparation of the text for this manuscript. TM assembled the tables and figures for the manuscript. All the authors contributed to the article and approved the submitted version.

## Conflict of Interest

The authors declare that the research was conducted in the absence of any commercial or financial relationships that could be construed as a potential conflict of interest.
